# Supplementary figures and images for: Characterization of the virome of shallots affected by the shallot mild yellow stripe disease in France
Source: PLoS One. 2019 Jul 24;14(7):e0219024. doi: 10.1371/journal.pone.0219024 (PMC6655591; doi:10.1371/journal.pone.0219024)

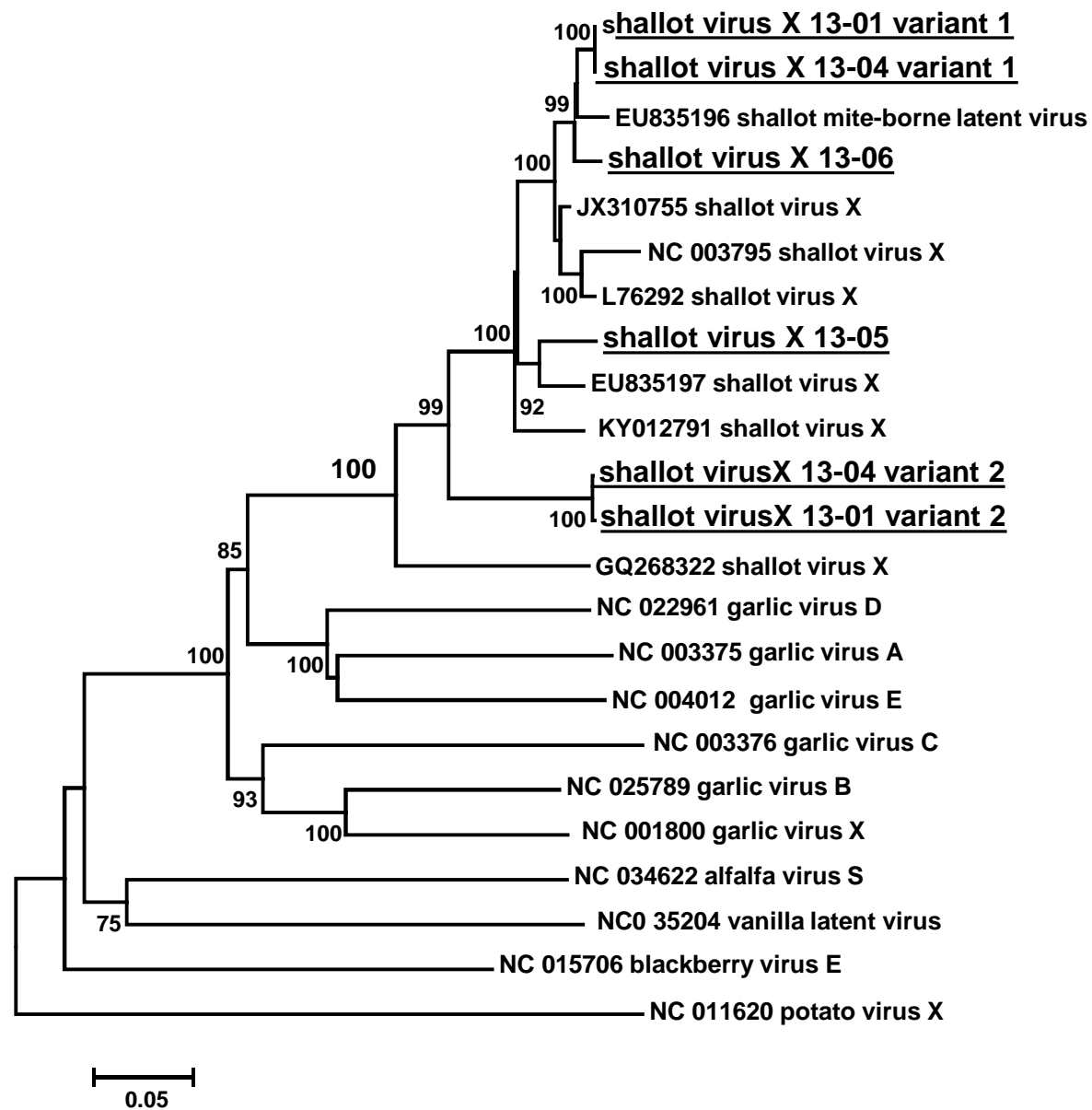

Supplement: S1 Fig — Validity of branches was evaluated by bootstrap analysis (1,000 replicates). Only bootstrap values above 70% are shown. The scale bar represents 5% amino acid divergence. The sequences of ShVX determined in this work are underlined. Potato virus X (NC011620, genus Potexvirus) was used as outgroup. (PDF) [file pone.0219024.s001.pdf]

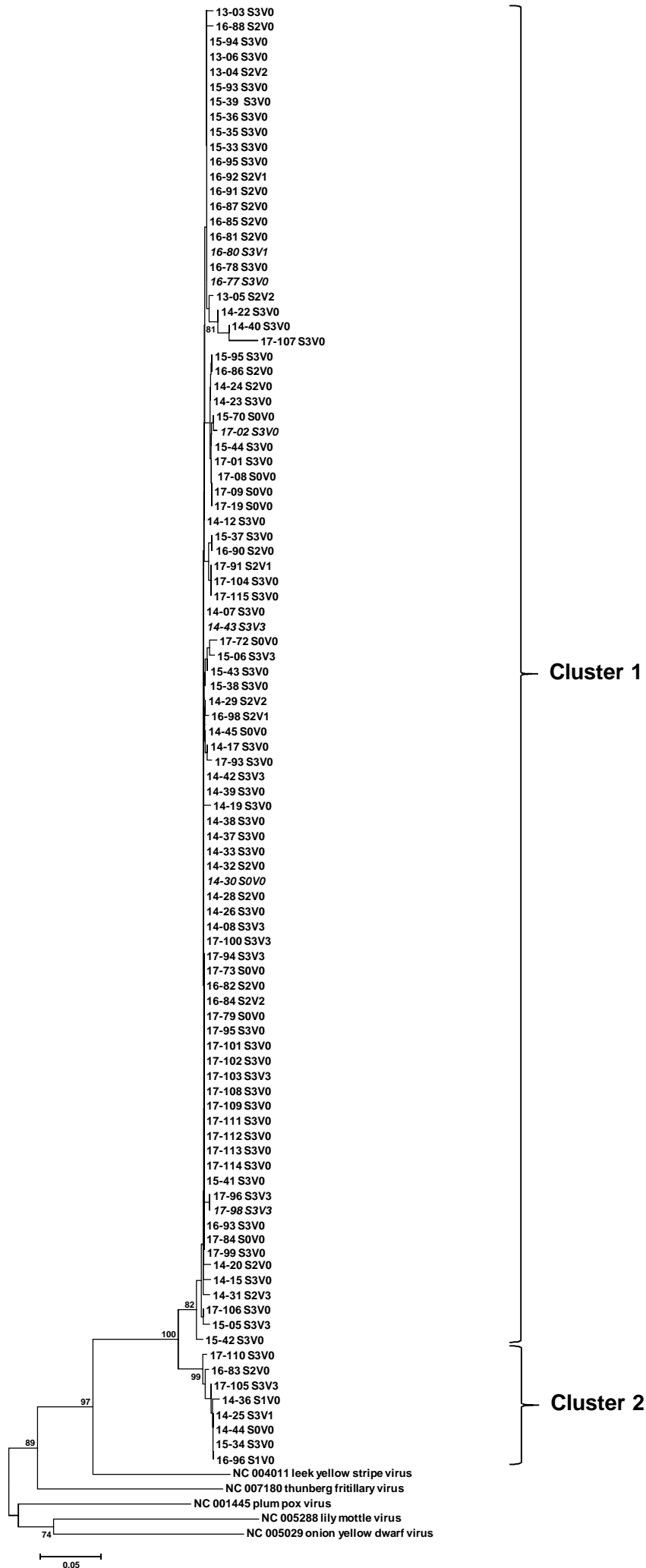

Supplement: S2 Fig — Statistical significance of the branches was evaluated by bootstrap analysis (1,000 replicates). Only bootstrap values higher than 70% are indicated. The scale bar represents 5% nucleotide divergence. The primer pair used for the RT-PCR (ShMYSV-F1/ShMYSV-R1) is indicated in S1 Table. Relevant nucleotide sequences were deposited in the GenBank database under accession numbers MG910501 to MG910598. Isolates found in co-infection with onion yellow dwarf virus or leek yellow stripe virus are indicated in italics. The scores of leaves striping (S) and loss of vigor (V) are indicated (scale of notation from 0 to 3). The two identified phylogenetic clusters are indicated on the right of the figure. (PDF) [file pone.0219024.s002.pdf]
