# Supplementary material for: Characterization of the virome of shallots affected by the shallot mild yellow stripe disease in France
Source: PLoS One. 2019 Jul 24;14(7):e0219024. doi: 10.1371/journal.pone.0219024 (PMC6655591; doi:10.1371/journal.pone.0219024)
Supplement: S1 Table — (DOCX) [file pone.0219024.s003.docx]

|  | Sequence 5’-3’ | Amplicon position (targeted region) |
| --- | --- | --- |
| SMYSaV ^a^ in sample 13-06 |  |  |
| SMYSaV-Race2 ^b^ | CTTGAATTGATTTTAGTCGTCATAA | 1-182 (5’ UTR and P1) |
| SMYSaV-LD1 ^c^ | ACACGGTTAGCGGTTAATTACC | 10250-10635 (3’ UTR) |
| SMYSaV-F1 | CGGACATGGGATTAGCTCGCTA | 9560-9806 (CP) |
| SMYSaV-R1 | TTGTTACCAAGACATCACTGCAGT |  |
| ShVS ^a^ in sample 13-05 |  |  |
| ShVS-Race1 ^b^ | AGTGAGGCACGTATGAAAAAGGGCTAAGGT | 1-278 (5’ UTR and replicase) |
| ShVS-LD1 ^c^ | CCCACTGATGCCGAGCGCATTGC | 7839-8384 (Nucleic acid binding protein and 3’UTR) |
| ShVX ^a^ in sample 13-06 |  |  |
| ShVX-F1 | TTAGATGATTTTGATGACCTG | 1746-1940 (Replicase) |
| ShVX-R1 | GCCTTTCAGTTGGTCGGTCAGA |  |
| ShVX-F2 | ATTGAGATTCTCAAGCTCCAC | 2508-2935 (Replicase) |
| ShVX-R2 | ACCACGATGTTAATGCTGTCAG |  |
| ShVX-F3 | TCTTCTTTGCTGCCTACATTT | 8432-8689 (Nucleic acid binding protein) |
| ShVX-R3 | ATGACACTCAAACAGTCAGAA |  |
| ShVX-LD1 ^c^ | CTTATATGATAACCATGTGTGC | 8624-8908 (Nucleic acid binding protein and 3’ UTR) |
| ShVX variant 2 in sample 13-04 |  |  |
| ShVX-div-Race1 ^b^ | CCATAGCTTTCTTTGATGCTGCTTGCGCA | 1-210 (5’ UTR and replicase) |
| ShVX-div-NRace1 ^d^ | GAGTAGCCGGCTTTTGTGTTCGGGTCGCT | 1-163 (5’ UTR and replicase) |
| ShVX-div-F1 | ATCTCAGATCTCGTCACTCCC | 3170-3397 (replicase) |
| ShVX-div-R1 | TGCCGGCCTAAATCCGTCAG |  |
| ShVX-div-F2 | TCGTCGAGTGCAGCGAACAG | 7415-7664 (Triple gene block 3 and CP) |
| ShVX-div-R2 | ATCCCCTGATGCATTCAAACGA |  |
| ShVX-div-LD1 ^c,^ | CCAATCATCACACATGGTTTCAACCAG | 8322-8908 (CP-ORF6) |
| SLV ^a^ in samples 13-02 and 13-06 |  |  |
| SLV-div-Race1 ^b^ | ACAAGCGGGATGAGAATGAGGCACG | 1-285 (5’UTR and replicase) |
| SLV-div-LD1 ^c^ | CCTATGCCCGAGCTCGTAGAGC | 8103-8362 (Nucleic acid binding protein and 3’UTR) |
| OYDV ^a, e^ |  |  |
| OYDV-F | CGTTTGTTTGGCCTGGATGGTAACG | 10214-10539 (CP and 3’ UTR) |
| OYDV-R | GTCTCYGTAATTCACGC |  |
| LYSV ^a, f^ |  |  |
| LY5P | AATCTCAACACAACTTATRC | 12-732 (5’UTR and P1) |
| LY2M | AGTACGTTGCCTGCTCTGTAG |  |

^a^ SMYSaV: shallot mild yellow stripe associated virus, ShVS: shallot virus S, ShVX: shallot virus X, SLV: shallot latent virus, OYDV: onion yellow dwarf virus, LYSV: leek yellow stripe virus

^b^ used in combination with UPM provided by the 5′ rapid amplification of cDNA ends kit (Takara Bio Europe/Clontech)

^c^ used in combination with LD primer (5’ CACTGGCGGCCGCTCGAGCATGTAC 3’) as described by Youssef et al. 2011

^d^ used in combination with NUP provided by the 5′ rapid amplification of cDNA ends kit (Takara Bio Europe/Clontech) in a nested PCR

^e^ The primers were designed from Majumder and Baranwal, 2014

^f^ The primers were designed from Yoshida et al. 2012
