# Supplementary material for: Characterization of the virome of shallots affected by the shallot mild yellow stripe disease in France
Source: PLoS One. 2019 Jul 24;14(7):e0219024. doi: 10.1371/journal.pone.0219024 (PMC6655591; doi:10.1371/journal.pone.0219024)
Supplement: S2 Table — (DOCX) [file pone.0219024.s004.docx]

| Potyvirus name ^a^ | Large ORF (nt / **aa**) | CP ^b^ (nt / **aa**) | Nia-Pro-Nib ^c^ (nt / **aa**) |
| --- | --- | --- | --- |
| Shallot potyvirus | na ^d^ | na ^d^ | 92.3 / **94.3** |
| Leek yellow stripe virus | 68.8 / **73.6** | 68.9 / **74** | 72.5 / **82.5** |
| Garlic virus 2 | na ^d^ | 66.6 / **71.7** | na ^d^ |
| Garlic mosaic virus | na ^d^ | 69.5 / **74.1** | na ^d^ |
| Onion yellow dwarf virus | 49.4 / **40.8** | 60.7 / **59.8** | 57.6 / **53.5** |
| Shallot yellow stripe virus | 48.7 / **39.4** | 60.7 / **58.6** | 56.2 / **53.8** |
| Plum pox virus | 53.7 / **48.1** | 68.9 / **57.5** | 60.7 / **60.4** |
| Japanese yam mosaic virus | 52.6 / **46** | 61.2 / **59.3** | 57.7 / **56.4** |
| Thunberg fritillary virus | 55.1 / **50.1** | 63.4 / **58.8** | 60.8 / **62** |
| Sweet potato virus G | 53.2 / **47.4** | 58.1 / **55.1** | 59.5 / **59.3** |
| Lily virus A | na ^d^ | 60.3 / **59.2** | 60 / **62.3** |
| Lily mottle virus | 54.4 / **50.0** | 51 / **56.9** | 60.1 / **61.7** |
| Pennisetum mosaic virus | 51.5 / **43.3** | 56.7 / **55.3** | 57.8 / **52.9** |

^a^ Sequences retrieved from GenBank are the same as in Fig. 3B. Shallot potyvirus, L28079

^b^ CP, coat protein

^c^ Nia-Pro-Nib region analyzed corresponds to the region of the 2,525 nt-long sequence available for shallot potyvirus (L28079).

^d^ not applicable
