# Supplementary material for: Characterization of the virome of shallots affected by the shallot mild yellow stripe disease in France
Source: PLoS One. 2019 Jul 24;14(7):e0219024. doi: 10.1371/journal.pone.0219024 (PMC6655591; doi:10.1371/journal.pone.0219024)
Supplement: S3 Table — (DOCX) [file pone.0219024.s005.docx]

|  | Replicase | | Coat protein | |
| --- | --- | --- | --- | --- |
|  | Nucleotide | Amino acid | Nucleotide | Amino acid |
| Shallot latent virus 13-02 ^b^ | 60.2 | 62.6 | 70.5 | 75.9 |
| Shallot latent virus 13-03 ^b^ | 60 | 62.4 | 70.5 | 75.9 |
| Shallot latent virus 13-06 ^b^ | 60.1 | 62.8 | 69.3 | 76.2 |
| Shallot latent virus | 59.9 | 61 | 69.6 | 76.5 |
| Elderberry carlavirus D | 47.8 | 41.7 | 52 | 49.1 |
| Nerine latent virus | 48.4 | 39.9 | 53.6 | 48.8 |
| Narcissus symptomless virus | 47.9 | 39.7 | 53.7 | 48.5 |
| Butterbur mosaic virus | 47.8 | 41.4 | 51.3 | 47.5 |
| Garlic common latent virus | 47.2 | 39.2 | 48.9 | 45.8 |

^a^ Sequences retrieved from GenBank are the same as in Fig. 4

^b^ The three isolates of shallot latent virus 13-02, 13-03, and 13-06 have been determined in this work
